# Supplementary figures and images for: AlphaFold2 modeling and molecular dynamics simulations of an intrinsically disordered protein
Source: PLoS One. 2024 May 13;19(5):e0301866. doi: 10.1371/journal.pone.0301866 (PMC11090348; doi:10.1371/journal.pone.0301866)

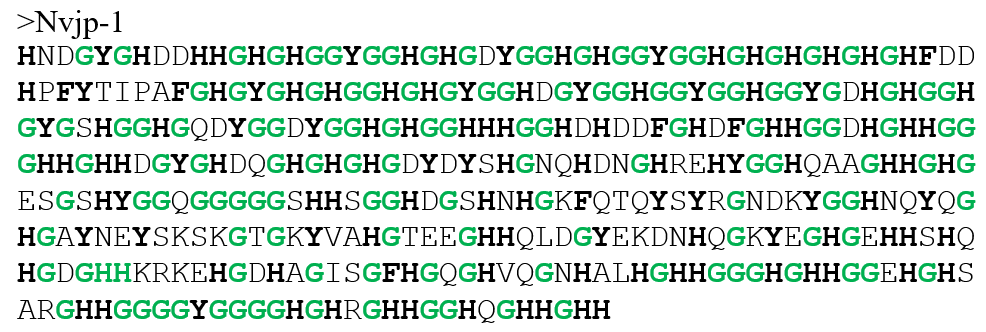

Supplement: S1 Fig — (TIF) [file pone.0301866.s001.tif]

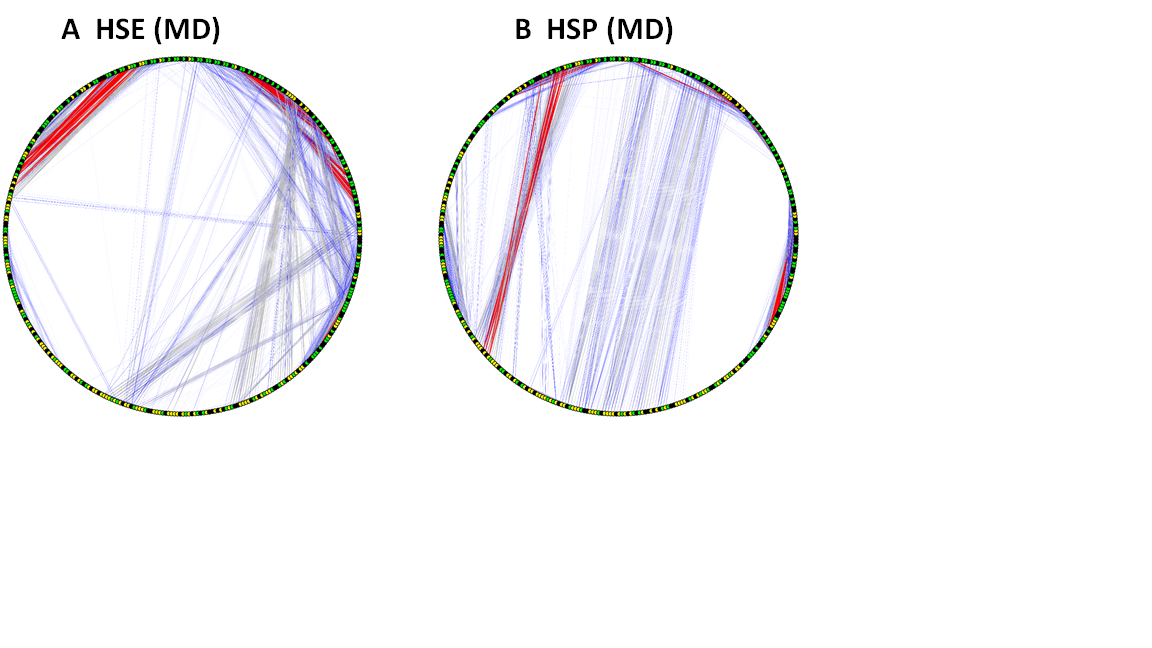

Supplement: S2 Fig — (TIF) [file pone.0301866.s002.tif]

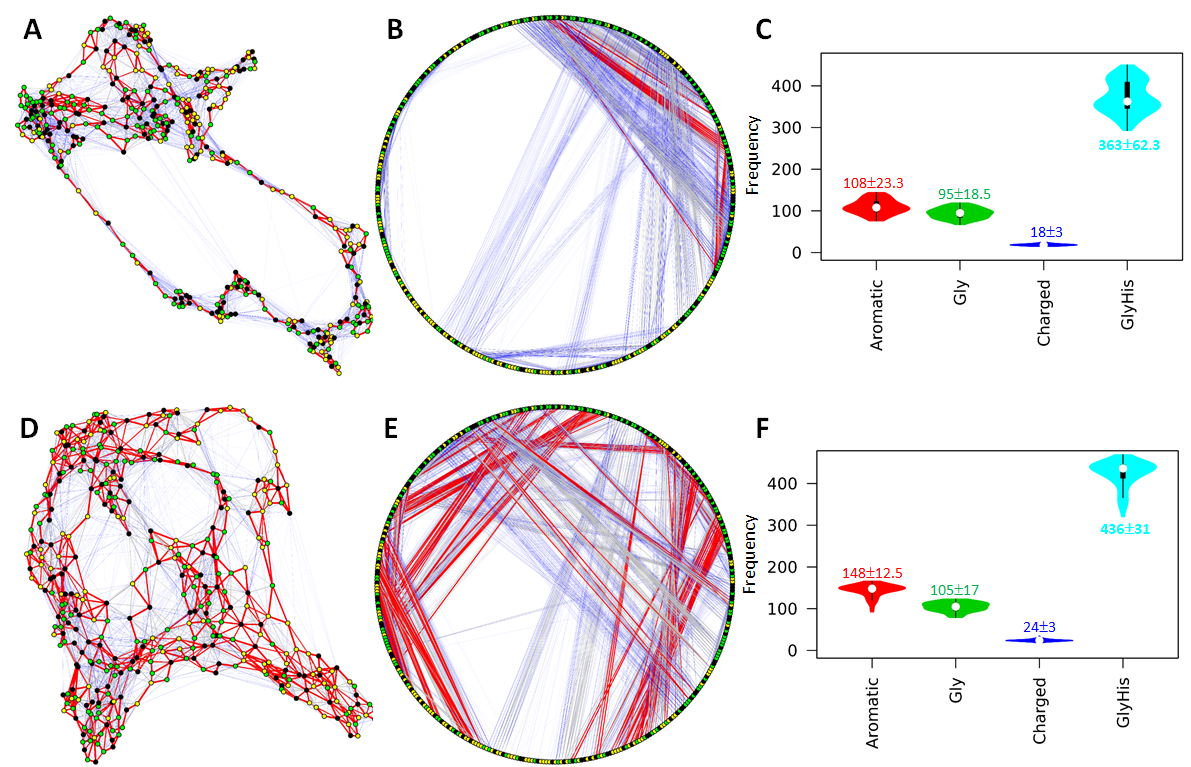

Supplement: S3 Fig — (TIF) [file pone.0301866.s003.tif]

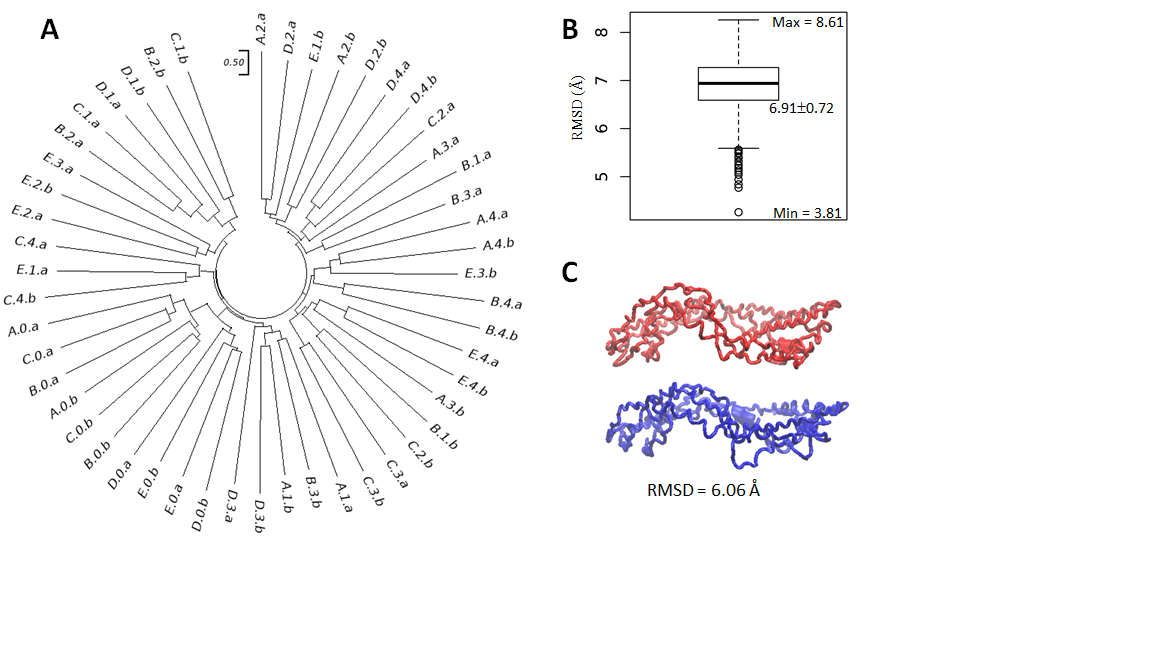

Supplement: S4 Fig — (TIF) [file pone.0301866.s004.tif]

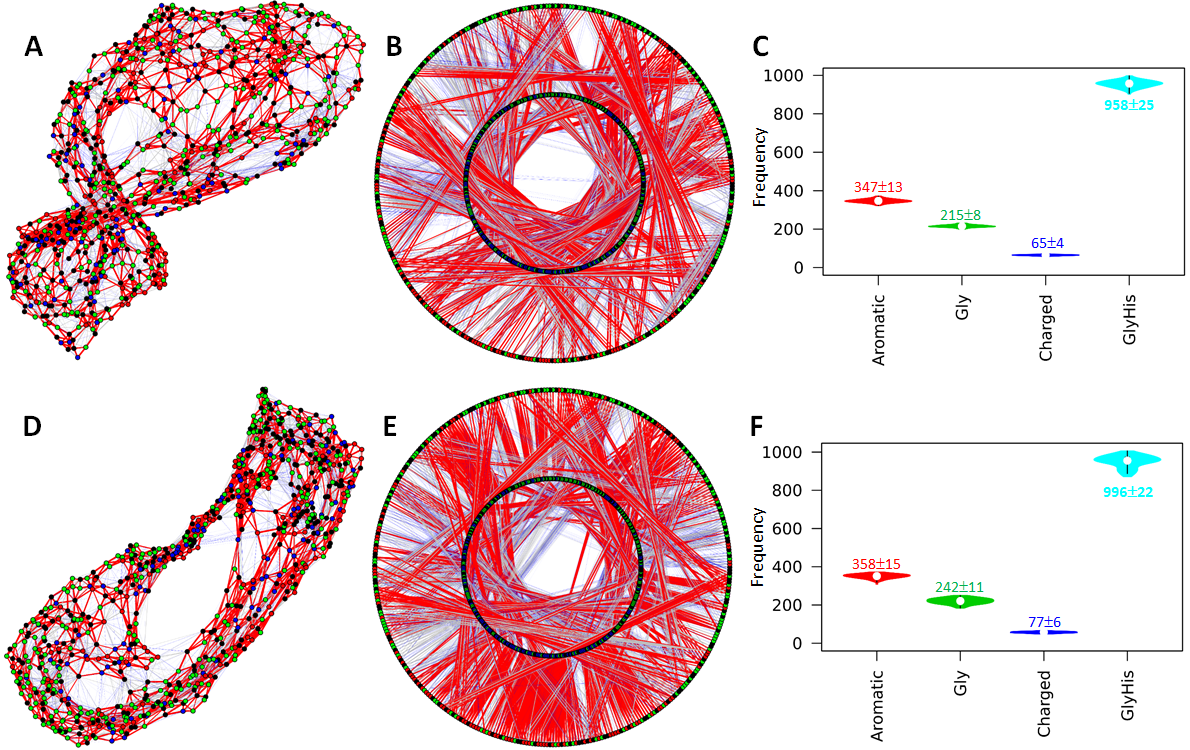

Supplement: S5 Fig — (TIF) [file pone.0301866.s005.tif]

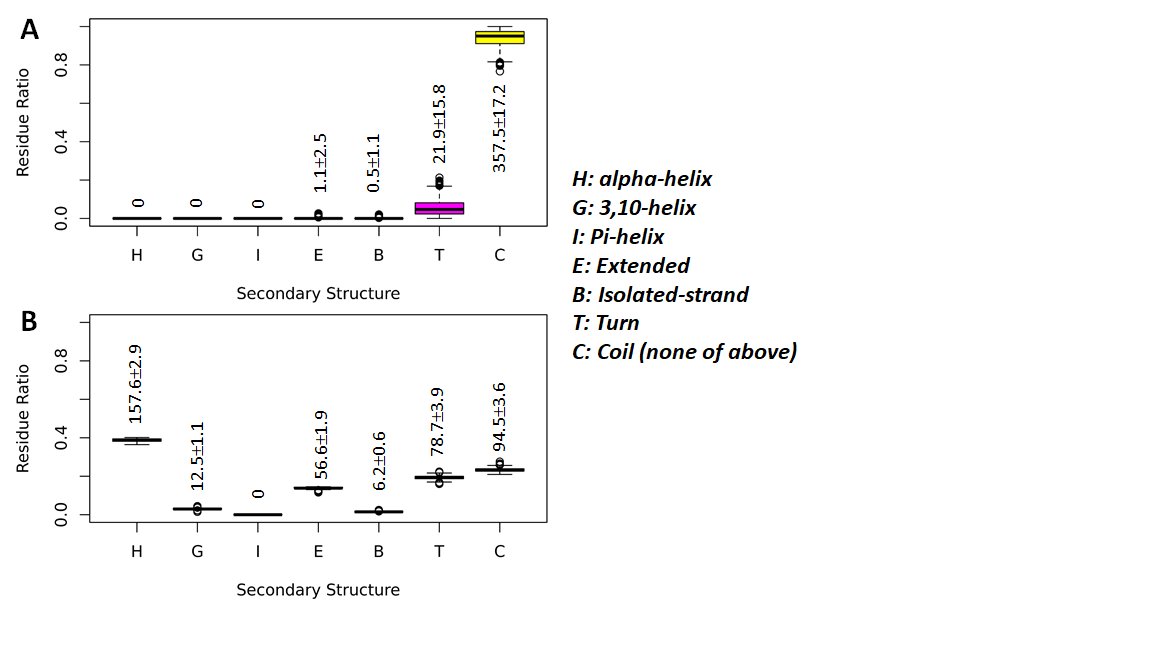

Supplement: S6 Fig — (TIF) [file pone.0301866.s006.tif]

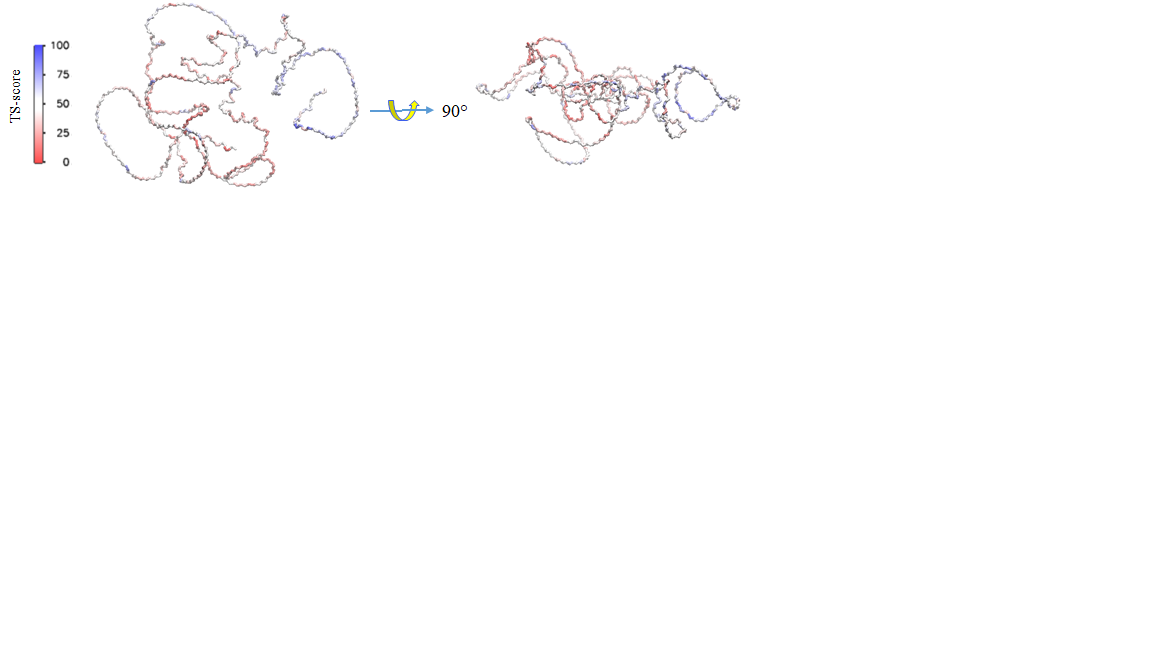

Supplement: S7 Fig — (TIF) [file pone.0301866.s007.tif]
