# Supplementary material for: AlphaFold2 modeling and molecular dynamics simulations of an intrinsically disordered protein
Source: PLoS One. 2024 May 13;19(5):e0301866. doi: 10.1371/journal.pone.0301866 (PMC11090348; doi:10.1371/journal.pone.0301866)
Supplement: Attachment — Submitted filename: Responses.docx [file pone.0301866.s008.docx]

When submitting your revision, we need you to address these additional requirements.

1. Please ensure that your manuscript meets PLOS ONE's style requirements, including those for file naming. The PLOS ONE style templates can be found at

https://journals.plos.org/plosone/s/file?id=wjVg/PLOSOne_formatting_sample_main_body.pdf and

https://journals.plos.org/plosone/s/file?id=ba62/PLOSOne_formatting_sample_title_authors_affiliations.pdf

***Response: Checked. The PLoS style of references have been used for this revision.***

2. Please note that PLOS ONE has specific guidelines on code sharing for submissions in which author-generated code underpins the findings in the manuscript. In these cases, all author-generated code must be made available without restrictions upon publication of the work. Please review our guidelines at https://journals.plos.org/plosone/s/materials-and-software-sharing#loc-sharing-code and ensure that your code is shared in a way that follows best practice and facilitates reproducibility and reuse.

***Response: Checked.***

3. We suggest you thoroughly copyedit your manuscript for language usage, spelling, and grammar. If you do not know anyone who can help you do this, you may wish to consider employing a professional scientific editing service.

Whilst you may use any professional scientific editing service of your choice, PLOS has partnered with both American Journal Experts (AJE) and Editage to provide discounted services to PLOS authors. Both organizations have experience helping authors meet PLOS guidelines and can provide language editing, translation, manuscript formatting, and figure formatting to ensure your manuscript meets our submission guidelines. To take advantage of our partnership with AJE, visit the AJE website (http://learn.aje.com/plos/) for a 15% discount off AJE services. To take advantage of our partnership with Editage, visit the Editage website (www.editage.com) and enter referral code PLOSEDIT for a 15% discount off Editage services. If the PLOS editorial team finds any language issues in text that either AJE or Editage has edited, the service provider will re-edit the text for free.

Upon resubmission, please provide the following:

The name of the colleague or the details of the professional service that edited your manuscript

A copy of your manuscript showing your changes by either highlighting them or using track changes (uploaded as a *supporting information* file)

A clean copy of the edited manuscript (uploaded as the new *manuscript* file)”

***Response: Checked.***

4. Thank you for stating the following in the Acknowledgments Section of your manuscript:

"This work was supported by funding from the OUSD (R&E) ARAP Program. The structural modeling and MD simulations were performed using the DoD HPC."

We note that you have provided funding information that is not currently declared in your Funding Statement. However, funding information should not appear in the Acknowledgments section or other areas of your manuscript. We will only publish funding information present in the Funding Statement section of the online submission form.

Please remove any funding-related text from the manuscript and let us know how you would like to update your Funding Statement. Currently, your Funding Statement reads as follows:

"This work was supported by funding from the OUSD (R&E) ARAP Program."

Please include your amended statements within your cover letter; we will change the online submission form on your behalf.

***Response: We added the Funding Statement section in this revision. Thank you!***

5. Please expand the acronym “OUSD (R&E)” (as indicated in your financial disclosure) so that it states the name of your funders in full.

This information should be included in your cover letter; we will change the online submission form on your behalf.

***Response: We expanded the acronym in this revision. Thank you!***

6. Thank you for stating the following financial disclosure:

"This work was supported by funding from the OUSD (R&E) ARAP Program."

Please state what role the funders took in the study. If the funders had no role, please state: ""The funders had no role in study design, data collection and analysis, decision to publish, or preparation of the manuscript.""

If this statement is not correct you must amend it as needed.

Please include this amended Role of Funder statement in your cover letter; we will change the online submission form on your behalf.

***Response: We added the suggested statement in this revision:***

***“This work was supported by funding from the Office of the Under Secretary of Defense for Research and Engineering (OUSD R&E), Applied Research for Advancement of S&T Priorities (ARAP) Program. The funders had no role in study design, data collection and analysis, decision to publish, or preparation of the manuscript.”***

***Thank you!***

7. We note that you have referenced ([32,33] observed a potential dimer band from SDS-PAGE, which can sustain high temperature in SDS detergent (unpublished).) which has currently not yet been accepted for publication. Please remove this from your References and amend this to state in the body of your manuscript: (ie “Bewick et al. [Unpublished]”) as detailed online in our guide for authors

http://journals.plos.org/plosone/s/submission-guidelines#loc-reference-style

***Response: We revised as your suggestion and cited the SDS-PAGE work as (Dennis et al., unpublished).***

***Thank you for this suggestion!***

8. Please include captions for your Supporting Information files at the end of your manuscript, and update any in-text citations to match accordingly. Please see our Supporting Information guidelines for more information: http://journals.plos.org/plosone/s/supporting-information.

***Response: We listed the “Supporting Information Appendix” at the end of the MS, before the references. Thank you!***

9. Please review your reference list to ensure that it is complete and correct. If you have cited papers that have been retracted, please include the rationale for doing so in the manuscript text, or remove these references and replace them with relevant current references. Any changes to the reference list should be mentioned in the rebuttal letter that accompanies your revised manuscript. If you need to cite a retracted article, indicate the article’s retracted status in the References list and also include a citation and full reference for the retraction notice.

***Response: Checked, thank you!***

[Note: HTML markup is below. Please do not edit.]

Reviewers' comments:

Reviewer's Responses to Questions

Comments to the Author

1. Is the manuscript technically sound, and do the data support the conclusions?

The manuscript must describe a technically sound piece of scientific research with data that supports the conclusions. Experiments must have been conducted rigorously, with appropriate controls, replication, and sample sizes. The conclusions must be drawn appropriately based on the data presented.

Reviewer #1: Yes

Reviewer #2: Yes

2. Has the statistical analysis been performed appropriately and rigorously?

Reviewer #1: Yes

Reviewer #2: Yes

3. Have the authors made all data underlying the findings in their manuscript fully available?

The PLOS Data policy requires authors to make all data underlying the findings described in their manuscript fully available without restriction, with rare exception (please refer to the Data Availability Statement in the manuscript PDF file). The data should be provided as part of the manuscript or its supporting information, or deposited to a public repository. For example, in addition to summary statistics, the data points behind means, medians and variance measures should be available. If there are restrictions on publicly sharing data—e.g. participant privacy or use of data from a third party—those must be specified.

Reviewer #1: Yes

Reviewer #2: Yes

4. Is the manuscript presented in an intelligible fashion and written in standard English?

PLOS ONE does not copyedit accepted manuscripts, so the language in submitted articles must be clear, correct, and unambiguous. Any typographical or grammatical errors should be corrected at revision, so please note any specific errors here.

Reviewer #1: Yes

Reviewer #2: Yes

5. Review Comments to the Author

Please use the space provided to explain your answers to the questions above. You may also include additional comments for the author, including concerns about dual publication, research ethics, or publication ethics. (Please upload your review as an attachment if it exceeds 20,000 characters)

Reviewer #1: The study utilizes various modeling and simulation techniques to explore the structural heterogeneity and disorder associated with Nvjp-1. The research delves into the unique characteristics of Nvjp-1, such as its modulated behavior by Zn-binding and its implications for dynamic sclerotization. Additionally, the study aims to understand the implications of disorder in the structural heterogeneity observed in the modeling and simulations of Nvjp-1.

- The manuscript is generally well-written and easy to follow. However, it would be beneficial to provide a clearer structure in the introduction section, outlining the main objectives and hypotheses of the study.

***Response: Thank you for this suggestion!***

***We have proposed the main objectives in the Introduction section, i.e., what defines an IDP. These include the sequence-based ID predictions, the structural heterogeneity from multiple AF2 models, and the distinct patterns in the residue-residue interaction network compared to other well-folded proteins. In the revision, we added a description paragraph at the end of the Introduction section to outline the structure of this manuscript.***

- Residue number should be mentioned for all the models in the main text

***Response: Thank you!***

***This work mainly discuss the intrinsically disordered protein, Nvjp-1, which has 381 AA, and a well-ordered protein, T7RdhA, which has 406 AA. The protein sequence of Nvjp-1 have been listed in the supporting information (Fig. S1). The residue number information can also be found in the Figure 7 caption. In this revision, we do added the amino acid numbers of the models in the main text.***

- Authors may calculate the one-dimensional (1D) distance variations (DV) between the Cɑ carbons to find the DV matrix from MD is highly consistent with the PAE matrix from AF2, indicating that the PAE matrix originates from protein dynamics

***Response: Thank you for this suggestion!***

***In many previous related modeling, including AF2 and RoseTTAFold, the distance matrices are usually measured using the distances between the Cβ carbons (Cα for Gly), as the main focuses are usually the functions and/or dynamics from the protein sidechains. We used this matrix in our previous analysis. However, we found this approach will lead to apparent errors. Please find the examples in Fig. S6 of the supplementary information in this paper:*** [***https://www.nature.com/articles/s41598-023-30310-x***](https://www.nature.com/articles/s41598-023-30310-x)***.***

***It is the main reason we think the approach used in the present work is reliable.***

- Suggest potential future directions for research based on the current findings. This could include experimental validations, further computational studies, or applications in drug discovery.

***Response: Thank you so much!***

***This is really a great suggestion! We changed Section 4 to “Conclusions and Future Directions”. We suggest that the recent advances, especially AF-cluster, can be applied to explore the protein folding spaces that may result in alternative insights to the protein functions; we also plan to use our approach in other directions including protein-ligand and protein-protein interactions, with the potential applications to drug discovery.***

Reviewer #2: Appreciable work has been carried out so far. However, the author can investigate the origin of Nvjp-1 from the phylogeny perspective in order to compare its closest protein sequence having an ordered form of arrangement that is meant for a specific function.

***Response: We thank you for this suggestion! We did mention in this paper that Nvjp-1 is an “orphan” protein with no closely related BLAST hits available. Therefore, we are not able to provide the phylogenetic insights of this protein so far. A deeper investigation on the phylogeny of Nvjp-1 or other IDPs, is out of the scoop of the present work. But we believe in the future, with the continuous development in both the protein sequences and structures, we might have the opportunity to complete this analysis to explore the functions and applications of the Nvjp-1 and Nvjp-1 related proteins.***

6. PLOS authors have the option to publish the peer review history of their aticle (what does this mean?). If published, this will include your full peer review and any attached files.

If you choose “no”, your identity will remain anonymous but your review may still be made public.

Do you want your identity to be public for this peer review? For information about this choice, including consent withdrawal, please see our Privacy Policy.

Reviewer #1: Yes: VINOD KUMAR YATA

Reviewer #2: No

[NOTE: If reviewer comments were submitted as an attachment file, they will be attached to this email and accessible via the submission site. Please log into your account, locate the manuscript record, and check for the action link "View Attachments". If this link does not appear, there are no attachment files.]

While revising your submission, please upload your figure files to the Preflight Analysis and Conversion Engine (PACE) digital diagnostic tool, https://pacev2.apexcovantage.com/. PACE helps ensure that figures meet PLOS requirements. To use PACE, you must first register as a user. Registration is free. Then, login and navigate to the UPLOAD tab, where you will find detailed instructions on how to use the tool. If you encounter any issues or have any questions when using PACE, please email PLOS at figures@plos.org. Please note that Supporting Information files do not need this step.
